# Substantial contribution of genetic variation in the expression of transcription factors to phenotypic variation revealed by eRD-GWAS

**DOI:** 10.1186/s13059-017-1328-6

**Published:** 2017-10-17

**Authors:** Hung-ying Lin, Qiang Liu, Xiao Li, Jinliang Yang, Sanzhen Liu, Yinlian Huang, Michael J. Scanlon, Dan Nettleton, Patrick S. Schnable

**Affiliations:** 1 Department of Agronomy, Iowa State University, 2035 B Roy J Carver Co-Lab, Ames, IA 50011-3650 USA; 2 Interdepartmental Genetics and Genomics Graduate Program, Iowa State University, Ames, IA 50011-3650 USA; 3 Department of Genetics, Developmental and Cellular Biology, Iowa State University, Ames, IA 50011-3650 USA; 4 The Broad Institute of MIT and Harvard, 75 Ames Street, Cambridge, MA 02142-1403 USA; 5 Department of Plant Sciences, University of California, Davis, CA 95616-5270 USA; 6 Department of Plant Pathology, Kansas State University, Manhattan, KS 66506-5502 USA; 7 Department of Plant Genetics & Breeding, China Agricultural University, Beijing, 100193 China; 8 DATA Biotechnology Beijing Co. Ltd, Beijing, 102206 China; 9 Plant Biology Section, Cornell University, Ithaca, New York 14850 USA; 10 Department of Statistics, Iowa State University, Ames, IA 50011-1210 USA; 11 Department of Agronomy and Horticulture, University of Nebraska, Lincoln, Nebraska 68583-0660 USA

**Keywords:** Transcription factors, Gene expression, GWAS, Phenotypes, Traits, Association studies

## Abstract

**Background:**

There are significant limitations in existing methods for the genome-wide identification of genes whose expression patterns affect traits.

**Results:**

The transcriptomes of five tissues from 27 genetically diverse maize inbred lines were deeply sequenced to identify genes exhibiting high and low levels of expression variation across tissues or genotypes. Transcription factors are enriched among genes with the most variation in expression across tissues, as well as among genes with higher-than-median levels of variation in expression across genotypes. In contrast, transcription factors are depleted among genes whose expression is either highly stable or highly variable across genotypes. We developed a Bayesian-based method for genome-wide association studies (GWAS) in which RNA-seq-based measures of transcript accumulation are used as explanatory variables (eRD-GWAS). The ability of eRD-GWAS to identify true associations between gene expression variation and phenotypic diversity is supported by analyses of RNA co-expression networks, protein–protein interaction networks, and gene regulatory networks. Genes associated with 13 traits were identified using eRD-GWAS on a panel of 369 maize inbred lines. Predicted functions of many of the resulting trait-associated genes are consistent with the analyzed traits. Importantly, transcription factors are significantly enriched among trait-associated genes identified with eRD-GWAS.

**Conclusions:**

eRD-GWAS is a powerful tool for associating genes with traits and is complementary to SNP-based GWAS. Our eRD-GWAS results are consistent with the hypothesis that genetic variation in transcription factor expression contributes substantially to phenotypic diversity.

**Electronic supplementary material:**

The online version of this article (doi:10.1186/s13059-017-1328-6) contains supplementary material, which is available to authorized users.

## Background

Many projects are underway to identify loci that contribute to traits, and the methods to do so remain under development [1]. Most commonly, associations are sought between genetic variants (e.g., SNPs) and variation in trait values via genome-wide association studies (GWAS). Typical approaches to GWAS exploit linkage disequilibrium (LD) between genetic variants such as SNPs and loci that directly affect traits of interest. There are two main approaches for identifying such associations, mixed linear models (MLM) and Bayesian-based approaches.

MLM solutions have been developed to overcome the confounding effects of population structure and the relatedness among individuals, and provide increased computational efficiency and statistical power [2–5]. Typical MLM solutions estimate effects based on single markers and require the use of covariances to account for population structure. However, these approaches for controlling for population structure also decrease statistical power [6]. In contrast, Bayesian methods apply multiple variable regression models combined with prior distributions and Markov chain Monte Carlo (MCMC) sampling to generate posterior distributions [7–9]. Meuwissen et al. [8] first proposed the methods of ridge-regression BLUP, BayesA, and BayesB. BayesB assumes marker effects have identical and independent univariate t-distributions and assume that a designated portion of markers have no effect. BayesC is similar to BayesB, but marker effects are assumed to have a common variance [10].

Genes with regulatory functions often exhibit high levels of expression variation across species [11, 12] compared to metabolism-related genes [13]. Several studies have revealed that, among primates, transcription factors (TFs) can evolve rapidly in response to selection [14–16]. Within species, genes exhibit different levels of variation in expression among individuals and alterations in the regulation of the expression of TFs can contribute to novel phenotypes [17], such as branching in maize [18] or pelvic loss in three-spined stickleback fish [19].

Because variation in the regulation of gene expression contributes to phenotypic diversity [20], efforts have been made to identify genetic variants associated with variation in transcript accumulation, i.e., expression quantitative trait locus (eQTL) analyses [21]. Genetic variants detected via eQTL analysis can act *in cis* or *in trans*. The relative contributions of *cis*- and *trans*-acting eQTLs on phenotypic variation are unknown. *Cis*-variation is often considered a key mechanism in creating phenotypic novelty [22] and contributes to adaptive evolution [23–25]. Indeed, *cis*-effects have played a major role on gene expression during the domestication of maize [22]. It is worth noting, however, that due to limitations in statistical power it is typically more difficult to detect *trans-*acting eQTLs than *cis-*acting eQTLs [26]. Even so, many *trans-*eQTLs have been identified in maize [26, 27] and other species [28, 29].

Maize is one of the most genetically and phenotypically diverse species [30] and has a rich collection of genetic resources [31], making it an important model system. Because maize exhibits high levels of SNP diversity and low LD, it exhibits high statistical power and resolution in GWAS [32]. We used this model species to test the role of variation in the expression of TFs and more generally variation in transcript accumulation on phenotypic variation. Following deep RNA-seq analysis of multiple tissues from diverse inbred lines we established that TFs are depleted among genes that exhibit high levels of expression variation across genotypes. Next, we developed a Bayesian-based statistical method for using RNA-seq measurements of transcript accumulation as the explanatory variables in GWAS and thereby directly demonstrate an association between variation in transcript accumulation of TFs and phenotypic variation for a diverse collection of traits.

## Results

RNA-seq was conducted on mRNA extracted from multiple maize organs (seedling shoot apex, immature unpollinated ears, immature tassels, seedling shoots and roots) collected from the 27 inbred founders of the nested association mapping (NAM) population. Six billion raw 101-bp reads were generated, trimmed, filtered, and aligned to the B73 reference genome (“Methods”); 2.9 billion non-stacking uniquely aligned reads were used to assay transcript accumulation levels (Additional file 1).

### Identification of genes that are variably or stably expressed across tissues

To identify genes that exhibit extreme levels of variation in transcript accumulation across tissues, a series of model selection procedures was performed. Ultimately, we selected negative binomial distributions to model the distributions of read counts for genes, and the scaled log of over-dispersion parameters of quasi-negative binomial generalized linear models to minimize the correlation between expression variation and expression levels (“Methods”). Henceforth, the scaled log10 over-dispersion parameters will be termed “variation in gene expression”.

Of 39,656 high-confidence “filtered-gene set” (FGS) genes, 29,609 have sufficient levels of transcript accumulation (“Methods”) to be used in subsequent analyses. The distribution of variation of gene expression across tissues was a left-skewed distribution (Additional file 2: Figure S1a). We defined the upper and lower 2.5% percentiles of this distribution as tissue variable expression (T-VE) genes (N = 741 genes) and tissue stable expression (T-SE) genes (N = 741) (Additional file 2: Figure S1a). TFs as a group were enriched among T-VE genes (*P* value = 0.008) and homeobox (*P* value = 0.03) and MADS box families of TFs (*P* value = 5 × 10^−5^) were specifically enriched among T-VE genes (“Methods”). In contrast, TFs were depleted among T-SE genes (*P* value = 0.0005; Additional file 2: Figure S1b and Additional file 3c).

### Identification of genes that are variably or stably expressed across genotypes

A similar approach was used to identify genotype variable expression and genotype stable expression (G-VE and G-SE) genes. The distribution of variation in gene expression across genotypes demonstrated a left-skewed distribution (Additional file 2: Figure S2a). Although TFs were enriched among genes that exhibited higher than median levels of variation in gene expression (*P* value = 3 × 10^−5^), TFs were underrepresented among both G-VE and G-SE genes (*P* values = 0.002 and = 0.046, respectively; Additional file 2: Figure S2b). Specifically, although 46 TFs would be expected among the G-VE genes by chance, only 22 were observed (Additional file 4). Similar results were obtained when the G-VE and G-SE genes were defined as being the upper and lower 5 and 10% of all genes.

*Arabidopsis thaliana* RNA-seq data generated by Kawakatsu et al. [33] (N = 727 genotypes) were analyzed using similar approaches. Consistent with our maize results, TFs were depleted among G-VE (*P* value = 1.0 × 10^−5^) and G-SE (*P* value = 0.004) genes in *Arabidopsis* (Additional file 2: Figure S3). As was observed for maize, *Arabidopsis* TFs were enriched among those genes that exhibit higher than median levels of expression variation across genotypes (*P* value = 4 × 10^−8^).

### Correlation of variation in maize gene expression across genotypes and tissues

A linear trend was observed between tissue-wise and genotype-wise variation in gene expression (*r* ^2^ = 0.64, *P* value ~ 2 × 10^−16^; Additional file 2: Figure S4). Based on whether a gene demonstrated stable or variable variation of gene expression across tissues and genotypes, maize genes could be classified into nine categories (Additional file 3).

The 520 T-VE genes that are neither G-VE nor G-SE are significantly enriched in TFs overall (*P* value = 9 × 10^−5^) and enriched in several specific TF families, including Homeobox/HOX (*P* value = 0.02), MADS (*P* value = 2.6 × 10^−5^), and Squamosa promoter binding protein (SPB; *P* value = 0.03) genes (Additional file 3e). Generally, HOX genes function in organ identity [34] and SBPs function in phase change [35]. In contrast, the 330 genes classified as being both T-SE and G-SE are depleted for TFs (*P* value = 0.006; Additional file 4).

### Expression read depth genome-wide association study

Based on the findings that TFs exhibited moderate variation in expression across genotypes, we were interested in testing the contribution of variation in transcript accumulation levels of TFs to phenotypic diversity. To directly test this association, we developed a Bayesian-based statistical approach for using transcript accumulation as the explanatory variable during GWAS.

Typically, a GWAS is conducted using SNP genotypes as explanatory variables. We reasoned that using transcript accumulation as an explanatory variable for GWAS would have certain advantages in that gene expression levels potentially integrate the effects from multiple loci that contribute to phenotype variation. To the extent that these hidden multiple locus effects poorly explained by single genotyped SNPs, expression read depth genome-wide association studies (eRD-GWAS) may better explain variation in trait values. eRD-GWAS also have the potential to integrate the effects of epigenetic variation that contributes to variation in gene expression and other traits. To test the hypothesis that variation in transcript accumulation can explain diversity in trait values that is missed by traditional GWAS, we analyzed a set of lines which had been both genotyped and phenotyped and for which RNA-seq data were available.

The SAM (shoot apical meristem) diversity panel consists 369 diverse inbred lines, including commercially relevant inbreds with expired plant variety protection (PVP) [36]. We have genotyped this panel with 1.28 million SNPs [36]. In addition, we conducted RNA-seq on apex tissue (which includes the SAM) from each of these inbreds [36]. Using these RNA-seq data we calculated RPKM values for each of the 39,656 FGS genes in the maize genome for each of the inbreds in the SAM panel.

Each of the inbreds in the SAM diversity panel had previously been phenotyped for multiple traits related to the shoot apical meristem [37], i.e., volume, height, parabola radius, arc length, and SAM surface area [36], and a variety of other traits, including the mean node number [36], ear height, and days to anthesis (DTA) [38]. During the current study we phenotyped these inbreds for five additional traits, i.e., stalk circumference, stalk cross-sectional area, maximum and minimum stalk diameter, and number of nodes with brace roots. These traits exhibit varying degrees of correlation (Additional file 2: Figure S5), some of which have been reported previously [39].

To test the hypothesis that eRD-GWAS can identify loci that contribute to variation in traits that are not identified by traditional SNP-based GWAS, we analyzed all five SAM-related and eight other traits using both SNP genotypes and RPKM values as explanatory variables (“Methods”).

**Fig. 1:**
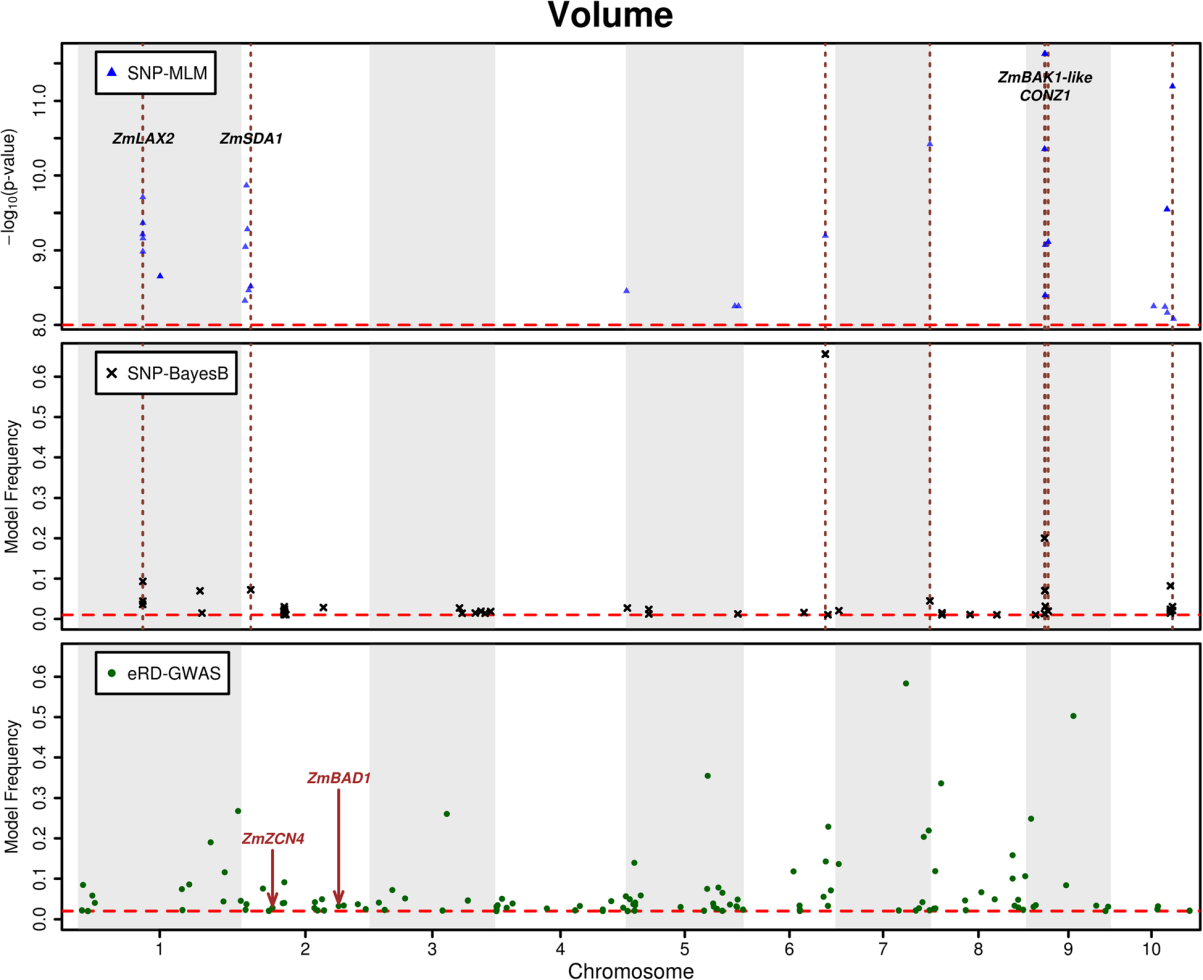
Manhattan plots of three types of GWAS results. The *upper panel* reports result from a SNP-based MLM implemented in GAPIT. Only signals with *P* values smaller than 1.0 × 10^−7^ are presented. The *middle* and *lower panels* report results from the SNP-based BayesB analysis and eRD-GWAS, respectively. The model frequency cutoffs for SNP BayesB and eRD-GWAS are 0.01 and 0.02, respectively (“Methods”). Overlapping associated SNPs in the upper two panels are indicated by *dashed lines*. Note not all overlapping SNPs can be distinguished in this plot. Gene IDs of some trait associated genes (“Methods”) are indicated

Typically, GWAS software that relies on MLMs is designed to use SNPs as the explanatory variables. We elected to use a BayesB-based approach to conduct eRD-GWAS in which RPKM values (expression data) served as the explanatory variables. Our rationale for selecting a Bayesian approach to GWAS is described in the “Methods”. The BayesB model is widely used in genomic selection. Instead of predicting phenotype, we used model frequency (the frequency with which a gene was included in a model) as a measure of the strength of the relationship between that gene’s expression pattern and the phenotype of interest. To validate the BayesB approach we repeated Leiboff et al.’s [36] SNP-based analysis of SAM volume using a MLM approach and in parallel conducted a SNP-based GWAS for SAM volume using a BayesB approach (“Methods”). As expected the results we obtained from our SNP-based GWAS using the MLM approach (Fig. 1) were very similar to those of Leiboff et al. [36]. The upper and middle panels of Fig. 1 provide results from the SNP-based MLM GWAS and the SNP-based BayesB GWAS. The 14 significant signals that overlap between the two approaches are indicated by vertical dashed lines on chromosomes 1, 2, 6, 7, 9, and 10. Nine of these 14 SNPs that were detected via both approaches are located in or near genes that have been shown previously to be associated with SAM volume [36]. If we consider SNPs present in the same genomic regions (to account for LD), 19 of the 54 SNPs detected by SNP-MLM were present in 30-kb windows centered on SNPs detected by SNP-BayesB. Similarly, 15 of 53 SNPs detected by SNP-BayesB were present in 30-kb windows centered on SNPs detected by SNP-MLM. These results established that the BayesB approach identified a significant subset of those SNPs identified by MLM GWAS, but that the BayesB approach also identified signals not identified by the MLM approach.

Based on these results we used BayesB-based eRD-GWAS to identify genes whose variation in transcript accumulation is associated with diversity in SAM volume. Approximately 500 genes (lower panel of Fig. 1) exceed the arbitrarily selected model frequency cutoff of 0.02 in the eRD-GWAS. If we search for candidate genes, GRMZM2G140721 is detected by both the SNP-based BayesB and eRD-GWAS. GRMZM2G140721 is a predicted transcriptional factor in *Arabidopsis*, rice, and maize. In total, 120 genes identified via eRD-GWAS (i.e., eRD genes) were not located within 30-kb windows centered on the chromosomal positions of SNPs identified via either SNP-based GWAS approach (MLM or Bayesian). Even so, some of these genes detected via eRD-GWAS but not by SNP-based GWAS have previously been demonstrated to affect the morphology of the SAM. For example, *ZEA CENTRORADIALIS4* (*ZCN4*) functions in the maintenance of indeterminate shoot meristem, thereby affecting the transition to an inflorescence meristem [40] and *BRANCH ANGLE DEFECTIVE 1* (*BAD1*) [40] is a TCP class II gene that is expressed in inflorescence meristems and lateral organs where it functions to promote cell proliferation.

All GWAS provide lists of genes that are hypothesized to be associated with traits of interest. To assay the accuracy of the gene–trait associations from eRD-GWAS we performed a series of analyses, including tenfold cross-validation, eQTL analyses of eRD-GWAS genes, tests for the enrichment of eRD-GWAS genes within specific nodes of RNA co-expression networks, protein–protein interaction networks, and gene regulatory networks.

### Tenfold cross-validation

Tenfold cross validation is a technique used for assessing the accuracy of prediction models [41]. Our tenfold cross-validation analyses of the results of eRD-GWAS (“Methods”) yielded accuracies of 0.41–0.76, indicating that eRD-GWAS accurately detects associations between variation in transcript accumulation and multiple traits (Additional file 5). Based on comparisons to similar cross-validation analyses conducted using results from SNP BayesB, the accuracies of the two approaches are similar for multiple traits (Additional file 5).

### eQTL for eRD-GWAS-detected genes (eRD genes)

If eRD-GWAS is accurately identifying genes that contribute to variation in a trait, we would expect that eQTLs that act *in trans* to regulate the expression of eRD genes may also be associated with variation in that trait. Hence, we conducted an eQTL analysis using an MLM approach (“Methods”) for the five eRD genes associated with the DTA trait that had the highest model frequencies. The resulting eQTLs were compared to the eRD genes and also to the genes associated with the DTA trait via BayesB GWAS (Additional file 6). Hypergeometric analyses (“Methods”) established that the eQTLs were enriched in genes associated with variation in the DTA trait. To ensure this phenomenon was robust across traits, we used the same strategy on multiple traits (Additional file 7). The results were consistent with our hypothesis, i.e., that the eQTLs associated with specific eRD genes are enriched overall in eRD genes. This result provides support for the view that eRD-GWAS accurately identifies genes whose expression is associated with variation in trait values.

### eRD-GWAS enriched in an RNA co-expression network

To enhance the power of this analysis we first constructed an RNA co-expression network using WGCNA [42] using the RNA-seq data from the SAM diversity panel. We then determined gene ontology (GO) terms that were enriched among the genes within specific modules of the co-expression network (Table 1). The modules that were enriched for eRD genes associated with the DTA trait were also enriched for a variety of GO categories. The “honeydew” module was enriched for the GO category “maintenance of floral meristem identity”, which would appear to be relevant to the DTA trait. Other modules were enriched for categories that the literature reported may be relevant to the DTA trait, such as “metal ion transport”, “response to nitrate”, and “NAD(P) metabolic” [43–45].

**Table 1:** GO enrichment tests of RNA co-expression modules containing multiple eRD genes for the DTA trait

Module name	GO term enrichment of module	Number of eRD genes within module (percentage of eRD genes in module)	Log2 odds ratio for eRD genes in module
Thistle3	Metal ion transport; transferring phosphorus-containing groups; ATP binding	20 (54.1%)	9.17^**^
Navajowhite2	NAD(P) metabolic	18 (34.6%)	8.03^**^
Firebrick4	Nitrate transport; magnesium ion binding	15 (35.7%)	8.38^**^
Palevioletred3	Terpene synthase; regulation of transcription; response to nitrate	6 (11.1%)	6.33^**^
Honeydew	Cell wall organization; maintenance of floral meristem	4 (11.1%)	6.92^**^

^**^ *P* value of enrichment test < 0.01

### eRD-GWAS in protein–protein interaction networks

**Fig. 2:**
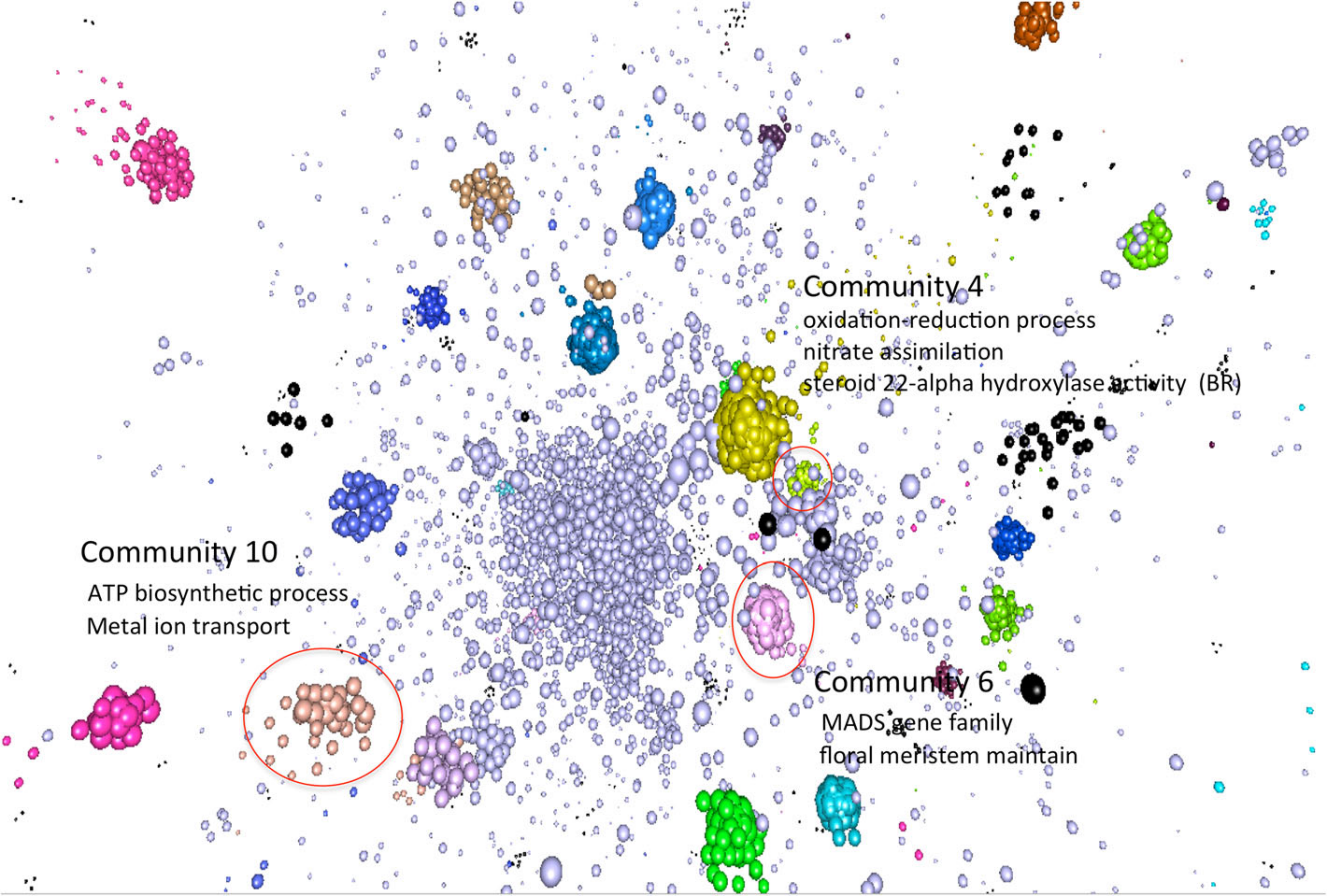
Visualization of a protein–protein interaction network that contains eRD genes. Highlighted communities that contain more than one eRD gene and in which eRD genes are statistically enriched are highlighted

Protein–protein interaction networks (PPINs) can be used to identify proteins (and genes) that contribute to phenotypes and thereby help elucidate complex genetic mechanisms [46]. We downloaded maize PPIN data from the maize PPIM [47], clustered proteins into network communities, and then tested whether eRD genes were enriched in network communities. As was the case for the enrichment tests within the RNA co-expression network, eRD genes were significantly enriched (“Methods”) in GO categories associated with the DTA trait among three of the 12 network communities that contained more than one eRD gene (Table 2 and Fig. 2). This finding provides further evidence that eRD-GWAS can identify biologically relevant gene–trait associations.

**Table 2:** GO enrichment among protein–protein interaction network communities that contain multiple eRD genes for the DTA trait

Community	GO term enrichment of community	Number of eRD genes within community percentage of eRD genes in community)	Log2 odds ratio for eRD genes in xcommunity
10	ATP biosynthesis process; metal ion transport	8 (7.41%)	4.75^**^
6	MADS-gene family; floral meristem maintain	5 (8.93%)	5.96^**^
4	Oxidation-reduction process; nitrate assimilation; steroid 22-alpha hydroxylase activity (BR)	12 (4.67%)	2.58^*^

^*^ *P* value of enrichment test < 0.05

^**^ *P* value of enrichment test < 0.01

### eRD genes in gene regulatory networks

**Fig. 3:**
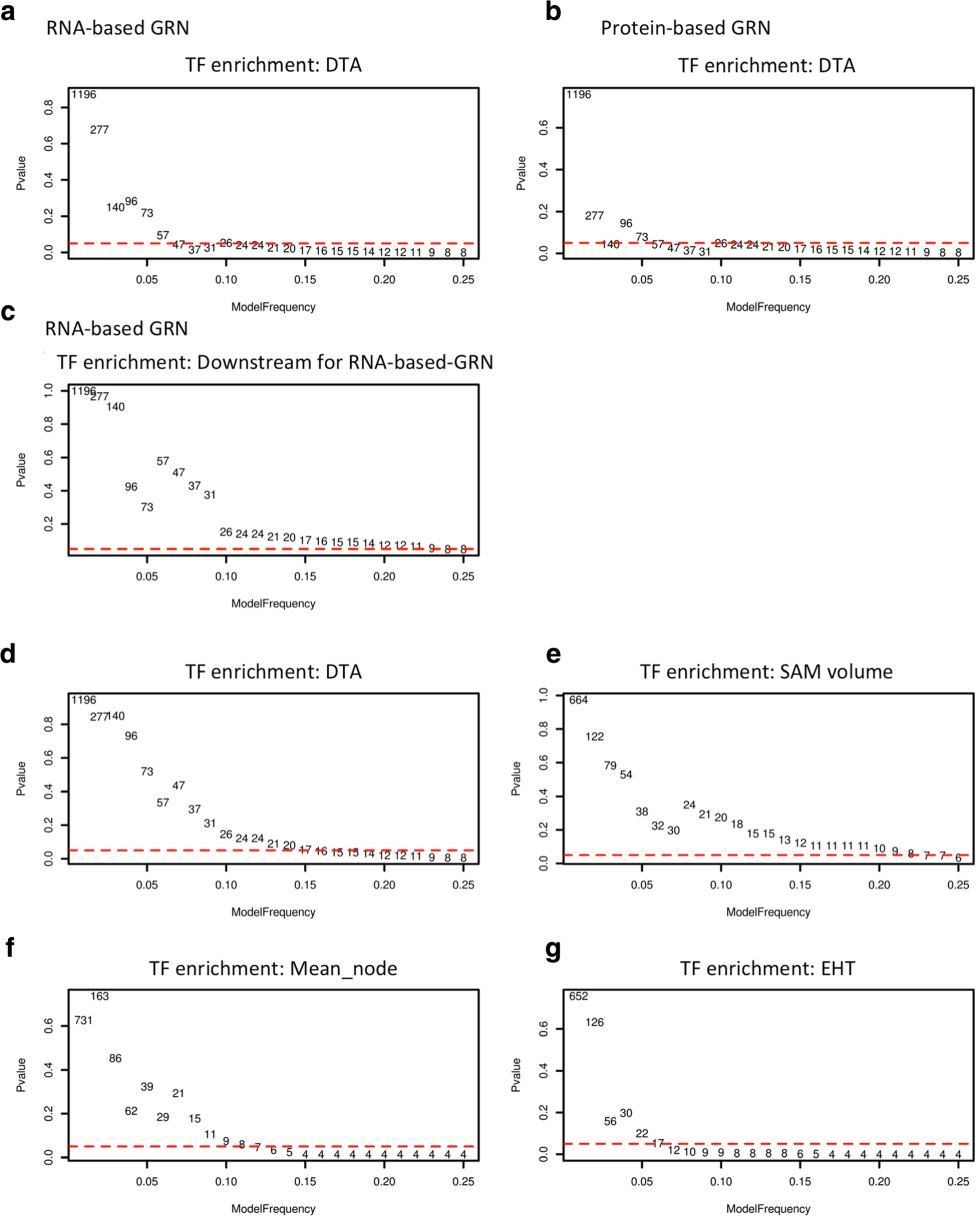
Enrichment testing for eRD genes. **a**–**c** Enrichment of “regulators” among eRD genes associated with the DTA trait at various model frequency cutoffs. **a**, **b** Numbers of eRD genes associated with the DTA trait that are defined as regulators of RNA- and protein-based GRNs by Walley et al. [48]. **c** Numbers of eRD genes that are themselves downstream of eRD genes that are regulators from the RNA-based GRNs. **d**–**g** Enrichment of TFs among eRD genes for various traits at various model frequencies. The number of eRD genes above indicated model frequency cutoffs are shown within each plot. The *red dashed lines* indicate *P* values of 0.05

Unlike co-expression networks, a gene regulatory network (GRN) is composed of directed edges that indicate biological relationships between pairs of nodes. For example, regulators are predicted to activate or suppress downstream genes. We examined the characteristics of our eRD genes within maize GRNs constructed using RNA-seq (23 tissues) or proteomic (33 tissues) data [48]; eRD genes were enriched among regulators in both the RNA- and protein-based GRNs (Fig. 3 a–c). Sets of eRD-GWAS genes selected using model frequency cutoffs larger than 0.03 have enrichment test *P* values smaller than 0.05, indicating that the targets of eRD-GWAS regulators are themselves enriched in eRD-GWAS genes (Fig. 3a–c). These results indicate that eRD-GWAS can identify both GRN regulators and their downstream targets.

### TFs are enriched among trait-associated genes from eRD-GWAS

As discussed earlier, TFs are enriched among genes that exhibit a higher than median level of variation in gene expression across genotypes. To test the hypothesis that the variation in expression of TFs affects phenotype, we conducted enrichment tests for TFs among eRD genes associated with 13 phenotypes using various model frequency cutoffs (Fig. 3d–g; Additional file 2: Figure S6). For 11/13 traits, as the stringency of model frequency cutoffs was increased, the enrichment of TFs among the eRD-GWAS genes also increased. This result demonstrates the importance of variation in the expression of TFs on phenotypic variation.

## Discussion

We were interested in comparing the variation in expression of TFs across tissues and genotypes to that of other genes. Using an RNA-seq data set derived from five tissues and 27 genotypes, we identified genes that exhibit low and high levels of expression variation across tissues (T-VE and T-SE genes) and genotypes (G-SE and G-VE). T-VE genes are enriched in TFs, and specifically enriched for Homeobox, MADS and Squamosa promoter binding (SPB) proteins. In contrast, T-SE genes are depleted for TFs.

In contrast to what was observed across multiple tissues, TFs were depleted among the G-VE and G-SE genes of both maize and *Arabidopsis*. Even so, in both species, TFs were enriched among those genes that exhibit higher than median levels of variation in gene expression. Interestingly, even though there is positive correlation between maize genes that exhibit high levels of expression variation across genotypes and tissues, TFs are not enriched among G-VE genes that are also T-VE. Based on these findings we hypothesize that extreme variation in expression of TFs across genotypes is constrained by selection against the extreme phenotypic variation that would be expected to arise via the action of TFs with extreme expression levels upon multiple downstream target genes. Similarly, because the NAM founders exhibit substantial phenotypic diversity, the depletion of TFs among the G-SE genes is consistent with a role of TFs in contributing to phenotypic diversity.

### Overview of eRD-GWAS

To test the hypothesis that variation in the expression of TFs (and other genes) across genotypes contributes to phenotypic variation, we developed eRD-GWAS, a statistical method that permits gene expression level to be tested as an explanatory variable during GWAS.

Using eRD-GWAS we detected several hundreds of trait-associated genes for each of multiple traits included in this study. The results of tenfold cross-validation indicated that the predicted phenotypes based on genes detected via eRD-GWAS are highly correlated with empirically measured phenotypes. In addition, many trait-associated genes have annotations consistent with their presumed roles in regulating the associated traits (Additional file 7). Hence, we concluded that the eRD-GWAS pipeline can successfully identify associations between variation in gene expression and diversity in phenotype. eQTL analyses of eRD-GWAS genes, tests for the enrichment of eRD-GWAS genes within specific nodes of RNA co-expression networks, PPINs, and GRNs provided further support for this conclusion.

### Challenges associated with GWAS

GWAS strategies identify genes that putatively contribute to variation in phenotypes. However, false positive results remain a challenge in GWAS [49]. The use of other types of genomic data in combination with SNP data has the potential to decrease biases and increase the power to detect true associations in GWAS. For example, efforts have been made to make use of eQTL results to increase the accuracy of GWAS [50, 51]. Although including eQTL results has the potential to decrease the rate of false positive associations, this approach can also result in elevated rates of false negative calls [49].

An alternative approach which we employed in this study is to use gene expression levels directly as explanatory variables for GWAS. This approach substantially reduces the multiple testing problem by using as explanatory variables expression data from ~ 40,000 maize genes vs. millions of available SNPs. This reduction in the number of explanatory variables also reduces the computational cost of eRD-GWAS compared to traditional SNP-based approaches.

Another group has shown that RNA expression patterns can predict human disease [20]. However, their statistical framework was intolerant of missing data which, required that transcriptomic data be imputed based on SNP data. This imputation would be expected to decrease accuracy. Further, their approach is limited to binary phenotypes (e.g., healthy vs. diseased). Jin et al. [52] also attempted to associate phenotypes with expression patterns. For a given gene, they classified lines as either being expressed or not based on RNA-seq data. Lines having intermediate levels of expression were treated as missing data. The conversion of continuous gene expression data into a binary classification scheme would be expected to decrease statistical power [53]. Because the data of Jin et al. were analyzed using an MLM approach, the limitations discussed in the “Background” apply. In contrast to the method of Jin et al., eRD-GWAS does not require that lines with intermediate expression levels be treated as missing data. In addition, our statistical framework is not limited to binary phenotypes as is the case for Gamazon et al. [20]. This is important because most important traits exhibit quantitative variation.

Because eRD-GWAS directly associates candidate genes with phenotypes, it eliminates the need to hunt for causative genes within windows surrounding trait-associated SNPs. One potential concern with eRD-GWAS is whether LD creates false trait associations between the expression of a gene that is simply linked to the causative gene. The Bayesian framework employed by eRD-GWAS functions to distinguish the effects of LD loci; our data suggest that this is in fact true not only for SNPs but also for expression data. For example, even though the expression patterns of various alleles of *ZmMADS69* are correlated with the expression patterns of other genes within the adjacent 1-Mb window (as well as genes across the genome), eRD-GWAS still could detect *ZmMADS69* as the gene with the highest model frequency for flowering time (Additional file 2: Figure S7).

Before using expression data to conduct eRD-GWAS, it is necessary to align RNA-seq reads to a reference genome. The substantial amount of SNPs [36] and structural polymorphism among maize haplotypes [54] may result in alignment biases that distort RPKM values and hence the power of eRD-GWAS. Although this bias did not interfere with our ability to detect trait-associated loci, the use of new alignment approaches that better control for polymorphisms [55] may provide additional power to eRD-GWAS.

This study included a direct comparison between the use of SNPs and expression data as explanatory variables within a common statistical framework. Our results establish that the two types of explanatory variables provide different association signals, such that some signals are detected by only one type of explanatory variable. This result argues that eRD-GWAS are complementary with SNP-based GWAS.

The Bayesian approach requires the selection of a model frequency cutoff which, unlike the q-value associated with MLMs, is in some sense arbitrary. If our selected model frequency cutoff (0.02) had been too relaxed, it is unlikely that strong statistical evidence for module-specific enrichment within the co-expression and PPINs would have been observed. Nor would we have been likely to observe a statistically significant enrichment of eQTLs for eRD genes among the eRD genes. If the selected model frequency cutoff were more stringent (i.e., if a larger model frequency), fewer genes would have been called as being associated with a given phenotype. This relationship is explored in Fig. 3 and Additional file 2: Figure S6, which demonstrate that the enrichment of TFs among the eRD genes for multiple traits is robust across a wide range of model frequency cutoffs, but that the enrichment *P* value can become more significant at increasingly stringent model frequency cutoffs. This finding is consistent with the hypothesis that a more stringent cutoff would result in a higher proportion of true positives, although presumably at the cost of more false negatives.

### Transcription factors contribute significantly to phenotypic variation

Variation in gene expression contributes to phenotypic variation [56] upon which natural and artificial selection can act. The mechanisms that regulate variation in gene expression can act *in cis* (e.g., transcription binding sites) or *in trans* (e.g., TFs). It has, for example, been shown that variants located upstream of maize genes are enriched in GWAS analyses of multiple morphological traits [57]. Similarly, GWAS signals are enriched near human TF binding sites [58]. These findings are at least consistent with the hypothesis that variation in TF binding sites contributes to phenotypic variation.

It is also likely that variation in the expression of TFs per se can contribute to phenotypic variation, and indeed specific cases of this type have been identified [18, 59]. Previous case studies have revealed roles for TFs in phenotypic evolution [60, 61]. In addition, genome-wide comparative genomics studies among primates have demonstrated that genes responsible for directional/diversifying selection are often TFs [11, 12, 62, 63]. As a step towards testing the hypothesis that TFs contribute substantially to phenotypic variation in maize, we demonstrated that TFs exhibit elevated levels of variation in expression across genotypes. More directly, using our newly developed eRD-GWAS method we established that genes associated with phenotypic variation for multiple traits are enriched in TFs, demonstrating that variation in the expression of TFs contributes substantially to phenotypic diversity in maize.

## Conclusions

TFs are enriched among genes with the most variation in expression across tissues and among genes with higher than median levels of variation in expression across genotypes. To better understand the relationship between variation in gene expression on phenotypes, we developed eRD-GWAS, which identifies associations between variation in gene expression and variation in phenotypes or traits. The enrichment of TFs among trait-associated genes identified via eRD-GWAS highlights the impact of expression variation on phenotypes. eRD-GWAS is complementary with SNP-based GWAS.

## Methods

### Tissue collection, library preparation, and RNA sequencing

Maize shoot apex, immature, unpollinated ears, immature tassels, and seedling shoots and roots of 27 NAM founders were collected for RNA extraction (Additional file 1). There exists a universal dilemma of sampling tissues from genotypes with different maturities. One must either sample from a common environment (same harvest date) and accept variation in developmental stage at harvest, or harvest at a common developmental stage and accept the risk of differences in micro-environment at harvest. For the NAM RNA-Seq experiment we elected to use the second approach.

Ear and tassel were harvested from greenhouse-grown plants with the exception of Ms71 ears, which were harvested from field-grown plants. Immature ear tips were harvested ~ 68 days after planting (depending on the maturity rate of each line). At this stage ear ranged from 0.5 to 3 inches; only the top one-third to one-fifth of each ear was collected. Tassels were harvested prior to tassel emergence, i.e., ~ 60 days after planting. Three healthy plants were sampled and pooled per genotype prior to homogenization in liquid nitrogen and RNA extraction. Maize shoot apexes were collected by pooling three to six 14-day-old seedlings from each NAM founder. Seedlings were grown by planting ten kernels of each line in germination paper which was rolled and placed in a tall plastic beaker filled with approximately 3 inches of tap water. Beakers were covered with “cling-wrap” and placed in a dark 28 °C incubator for approximately 4–5 days, when shoots emerged from the germination paper. Two to three inches of the shoot and root were cut and frozen in liquid nitrogen for immediate homogenization and extraction. Samples from three plants of each inbred were pooled for homogenization. For the SAM diversity panel, all plants were grown and sampled according to as in Leiboff et al. [36].

All RNA extractions were performed with the Qiagen RNeasy kit according to the manufacturer’s protocol. RNA was eluted twice with 30 μl RNase free water. Indexed RNA-seq libraries were prepared using the Illumina protocol outlined in the “TruSeq RNA Sample Preparation Guide” (part number 15008136 rev. A, November 2010). Maize shoot apex RNA was sequenced with an Illumina Genome Analyzer II instrument while ear, root, shoot, and tassel RNA were sequenced with an Illumina HiSeq 2000 instrument.

### RNA-seq reads: processing, alignment, and SNP calling

Quality trimming, alignment to the B73 reference genome, and SNP calling were as described by Leiboff et al. [36].

Alignment coordinates of confidently (uniquely) mapped reads within the same chromosomal regions were compared for potential read stacks caused by PCR artifacts during sequencing. If a stack consisting of two or more reads with identical start and end positions were detected, only a single read with best alignment score (least number of mismatches and least number of ambiguous bases) was selected for variant detection. If the distance from the left base pair to right base pair was more than 12,000 bp, the reads/read pairs were further removed. Reads with non-canonical splice sites were also removed.

### Discovery and annotation of expression variable/stable genes

Read counts are discrete and usually exhibit correlation between mean and variance [64]. Proper models, techniques, and summary statistics are essential to evaluate expression variation. To reduce ascertainment bias between expression level and expression variability, Pearson correlations were computed between expression level and each of several summary statistics (Additional file 2: Figure S8), including over-dispersion parameter of the Poisson model [65], mean coefficient of variance based on the Poisson model, deviance of the negative binomial model [66], and the over-dispersion parameter of the quasi-negative binomial model [65]. The R packages edgeR (version 3.14.0) [67] and QuasiSeq (version 1.0-8) [65] were used to estimate dispersion parameters and over-dispersion parameters of quasi-negative binomial GLMs (some graphical display used ggplot2, version 2.2.1 [68]). Full models were fitted when comparing Poisson, negative binomial, and quasi-negative binomial GLMs, as follows:

$$ \mathit{\log}\left({\lambda}_{ijk}\right)=\mu +{\alpha}_i+{\beta}_j+{o}_{ijk} $$

Where *λ*_*ijk*_ is mean fragment count for genotype i, tissue j, and observation k, *μ* is an intercept parameter, *α*_*i*_ is an effect of genotype i, *β*_*j*_ is an effect of tissue j, and *o*_*ijk*_ is the normalization offset for genotype i, tissue j, and observation k.

Of the four measures of variation discussed above, the over-dispersion parameter of quasi-negative binomial model, which measures the deviation of a gene’s read counts from the best-fitting negative binomial distribution, had the smallest correlation with expression level, and was thus used to measure expression variability (Additional file 2: Figure S8). The over-dispersion parameter Φ of quasi-negative binomial GLMs is:

$$ \Phi =\frac{Var(Y)}{\kappa E{(Y)}^2+E(Y)} $$

where Y is fragment count for a gene, Var(Y) and E(Y) are the variance and expectation of Y, respectively, and *κ* is the dispersion parameter of a negative binomial GLM. Tissue-wise over-dispersion parameters were estimated with genotype as the only factor in the model, while genotype-wise over-dispersion parameters were estimated treating tissue as the only factor in the model. A total of 29,609 genes with mean read counts ≥ 5 and numbers of samples with zero read counts ≤ 2 were included in the analysis. Z-score normalization was performed against log transformed over-dispersion parameter estimates, where:

$$ \mathrm{Z}=\frac{\mathit{\log}\left(\widehat{\varPhi}\right)-\widehat{E}\left(\mathit{\log}\left(\varPhi \right)\right)}{\sqrt{\widehat{Var}\left(\mathit{\log}\left(\varPhi \right)\right)}} $$

Upper and lower 0.025 quantiles of transformed normalized distributions were used to define highly variable and highly stable genes. MAPMAN annotation of maize filter gene sets (5b.61) was used to perform functional enrichment tests [69]. Fisher exact test was performed with the Benjamini–Hochberg method controlling false discovery rates (FDRs).

### Collection of phenotypic data

Phenotypic trait data were collected from a panel of 369 diverse inbreds designated as the “SAM panel” [36]. Data were collected from three plants per location in two fields grown in Ames, Iowa during the summer of 2014. Prior to data collection leaf sheaths and brace roots (if present) were removed. Measured traits included maximum and minimum stalk diameters, stalk circumference, stalk cross-sectional area, total node number, and number of nodes with brace roots (Additional file 8). Additional data from the SAM panel (or members of it) were obtained from the literature. For example, several traits associated with the SAM, including SAM height, radius, surface area, volume, and arc length from P1 notch to apex, were obtained from [36]. Ear height and DTA data were obtained from [38]. Phenotypic regression and phenotypic density distributions were conducted using the R “corrgram” package version 1.10 [70].

### Mixed linear model GWAS

GAPIT version 3.35 [71] was used for MLM GWAS. The model implemented in GAPIT was:

$$ \mathrm{y}=\mathrm{W}\upnu +\mathrm{X}\upbeta +\mathrm{Z}\upupsilon +\mathrm{e} $$

where y is the phenotype value, ν and β are unknown fixed effect vectors, and υ is a vector of random effects that follows a multivariable normal distribution with a null mean and a covariance matrix of G. G = K*σ* ^2^_a_, where K is the kinship matrix [2]. e follows a normal distribution with null mean and *σ* ^*2*^_*e*_ ***I*** variance. In general W, X, and Z are the matrices containing principal component scores that account for population structure, known covariates, and SNP genotypes, respectively. In our case, W contains scores for the first three principal components, X was not used because we had no known covariates to adjust for, and Z had data on 1.28 million SNPs. Manhattan plots were generated from our in-house R scripts based on the *P* value from the GAPIT results. The cutoff was arbitrarily set at 10^−7^. Other settings followed GAPIT’s defaults.

### Bayesian-based GWAS

We selected a Bayesian approach for exploring the relationship between gene expression and phenotype, rather than a MLM approach, for two major reasons. First, the multivariate Bayesian framework internally controls for the effects of other genes by testing whether the inclusion of a given marker (i.e., the expression level of a given gene) can explain more genetic variance in each MCMC (Markov chain Monte Carlo) iteration. Although it may be possible to fit all the markers (i.e., gene expression levels of all genes) simultaneously by iterating an MLM approach, this would be time consuming. In contrast, this feature is “baked into” the Bayesian approach. Equally important, population structure can be controlled automatically via Bayesian approaches that include multiple genes in each MCMC iteration [72]. In contrast, population structure information is required to control false positives as covariances in MLM, which can decrease statistical power.

Multiple genomic selection models were constructed employing different values of π (the proportion of SNPs, assumed to have no effect on phenotype). The accuracies of these various models were evaluated using tenfold cross-validation and heritability. We selected for each phenotype a value of π that yielded the maximum accuracy based on tenfold cross validation that has a heritability that is not so high as to raise concerns of over-fitting. This had the effect of thinning the number of predictors, resulting in a more limited number of descriptors, similar to the output of GWAS. Our approach differs from MLM GWAS in that rather than using a *P* value to reflect the strength of the relationship between a marker and a phenotype, we used the model frequency (the frequency with which a gene was included in a model) to reflect the strength of the relationship between that gene’s expression and the phenotype of interest.

The Bayesian-based GWAS was constructed using GenSel v4.1 [10] BayesC and BayesB methods. The model in GenSel was:

$$ \mathrm{y}=\mathrm{X}\upbeta +\mathrm{Z}\upupsilon +\mathrm{e} $$

where X, Z, β, and υ are the same as in the MLM model, e follows a normal distribution with null mean, and covariance matrix *σ* ^*2*^_*e*_ ***R*** (***R*** is a diagonal matrix), *σ* ^*2*^_*a*_, and *σ* ^*2*^_*e*_ have independent inverse Chi-square priors with degree of freedom 4 and scale parameters set to 50% of phenotypic variation as prior. For BayesB (eRD-GWAS) and BayesC (SNP-based GWAS), the fraction (f) of markers having no effect was set at 0.9996 and 0.995, respectively. We used a chain length of 41,000 and discarded 1000 iterations as a burn-in run. Significance cutoffs for SNP-BayesB and eRD-GWAS were set as model frequencies of 0.01 and 0.02, respectively. Then we used genetic variance and error variance posteriors from BayesC as priors in BayesB; other settings were as above. The accuracy of Bayesian-based GWAS results were estimated via tenfold cross-validation.

### Cross-validation, enrichment tests, network visualization, and GO enrichment

Tenfold-cross validations were conducted using the R “cvTools” package version 0.3.2 [73]. Enrichment test *P* values were based on hypergeometric distributions. Network visualization was conducted using “MANGO” software version 1.20 [74]. Clustering was conducted using the fastgreedy community method [75]. GO term enrichment analyses were conducted using the GOseq package version 1.20.0 [76]. Functional enrichment tests were based on MAPMAN annotations. The list of TFs used in the enrichment tests were obtained from the “Grassius database” [77]. A list of *Arabidopsis thaliana* TFs was downloaded from “AGRIS” [78]. *P* values for TF enrichment were obtained from single-tailed Fisher tests.

## Additional files

Additional file 1: Table S1. Summary of RNA-seq processing. (XLSX 64 kb)

Additional file 2: Supplemental figures. (PDF 5961 kb)

Additional file 3: Table S2. Functional enrichment and GO term enrichment tests. **a** Functional enrichment tests of genes that exhibit extreme expression variation across genotypes. **b** GO term enrichment tests of genes that exhibit extreme expression variation across genotypes. **c** Functional enrichment tests of genes that exhibit extreme expression variation across tissues. **d** GO term enrichment tests of genes that exhibit extreme expression variation across tissues. **e** Functional enrichment tests of genes that exhibit extreme expression variation across genotypes and tissues. **f** GO term enrichment tests of genes that exhibit extreme expression variation across genotypes and tissues. (XLSX 61 kb)

Additional file 4: Table S3 Enrichment test for TFs among nine different gene categories. (XLSX 35 kb)

Additional file 5: Table S4. Accuracy of associations from SNP-BayesB and eRD-GWAS as estimated via tenfold cross-validation. (XLSX 41 kb)

Additional file 6: Table S5. Characterization of five genes associated with DTA identified via eRD-GWAS with highest model frequencies. (XLSX 39 kb)

Additional file 7: Table S6. Comparison of eQTL analyses for 13 traits and eRD genes with different GWAS results. (XLSX 43 kb)

Additional file 8: Table S7. Phenotypes analyzed in this study. (XLSX 38 kb)

## Abbreviations

DTA: Days to anthesis
eQTL: Expression quantitative trait locus
eRD-GWAS: Expression read depth GWAS
FGS: Filtered-gene set
GLM: General linear model
GO: Gene ontology
GRN: Gene regulatory network
G-SE: Genotype stable expression
G-VE: Genotype variable expression
GWAS: Genome-wide association study
LD: Linkage disequilibrium
MCMC: Markov chain Monte Carlo
MLM: Mixed linear model
NAM: Nested association mapping
PPIN: Protein–protein interaction network
RPKM: Reads per kilobase of transcript per million mapped reads
SAM: Shoot apical meristem
SNP: Single-nucleotide polymorphism
SPB: Squamosa promoter binding protein
TF: Transcription factor
T-SE: Tissue stable expression
T-VE: Tissue variable expression
