# Supplementary material for: Substantial contribution of genetic variation in the expression of transcription factors to phenotypic variation revealed by eRD-GWAS
Source: Genome Biol. 2017 Oct 17;18:192. doi: 10.1186/s13059-017-1328-6 (PMC5645915; doi:10.1186/s13059-017-1328-6)
Supplement: Supplementary file 2 — Supplemental figures. (PDF 5961 kb) [file 13059_2017_1328_MOESM2_ESM.pdf]

Supplemental Materials for: Substantial contribution of genetic variation in the expression of transcription factors to phenotypic variation revealed by eRD-GWAS

SUPPLEMENTAL FIGURES:

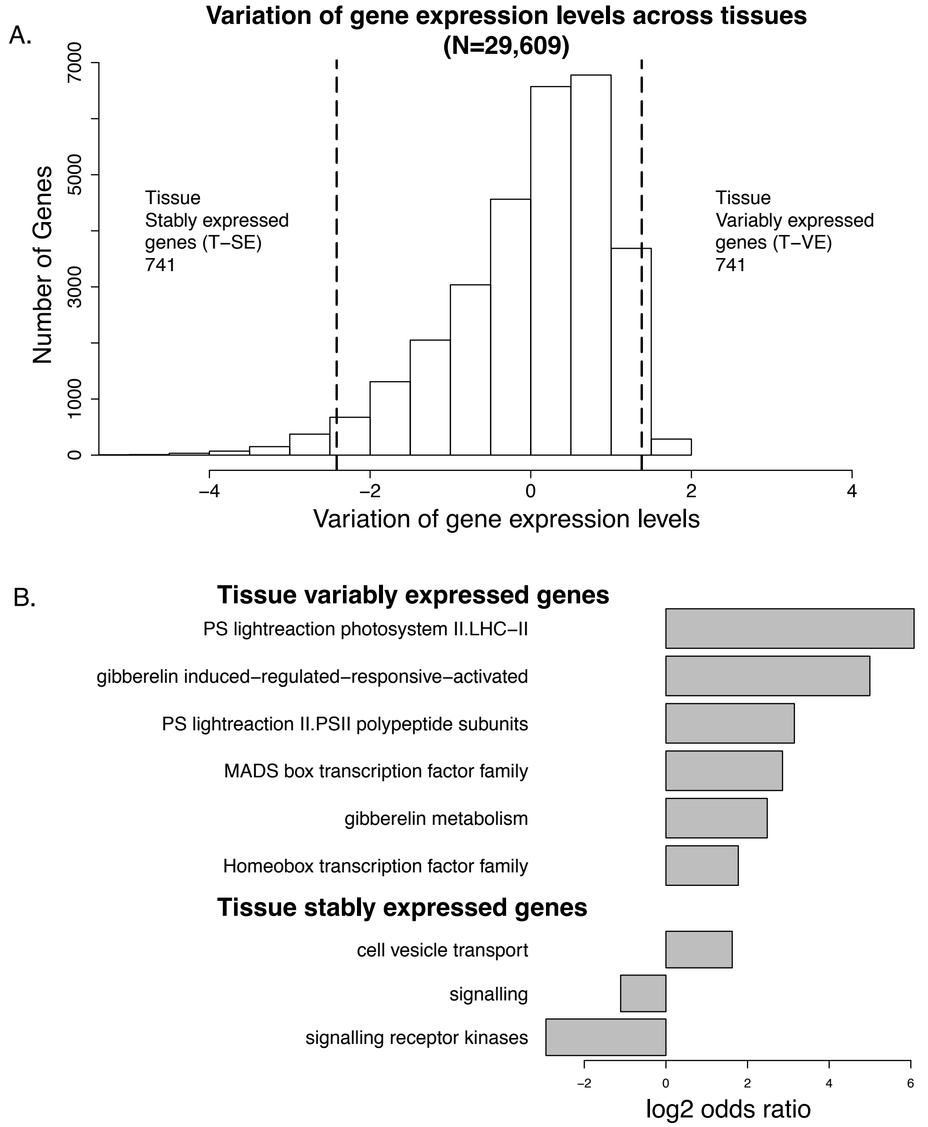

Figure S1. Identification and functional enrichment of T-VE and T-SE genes. a) Histogram of expression variation across tissues; dashed line corresponds to upper and lower 2.5% percentiles of the distribution. b) Functional enrichment of significant categories within T-VE and T-SE genes.

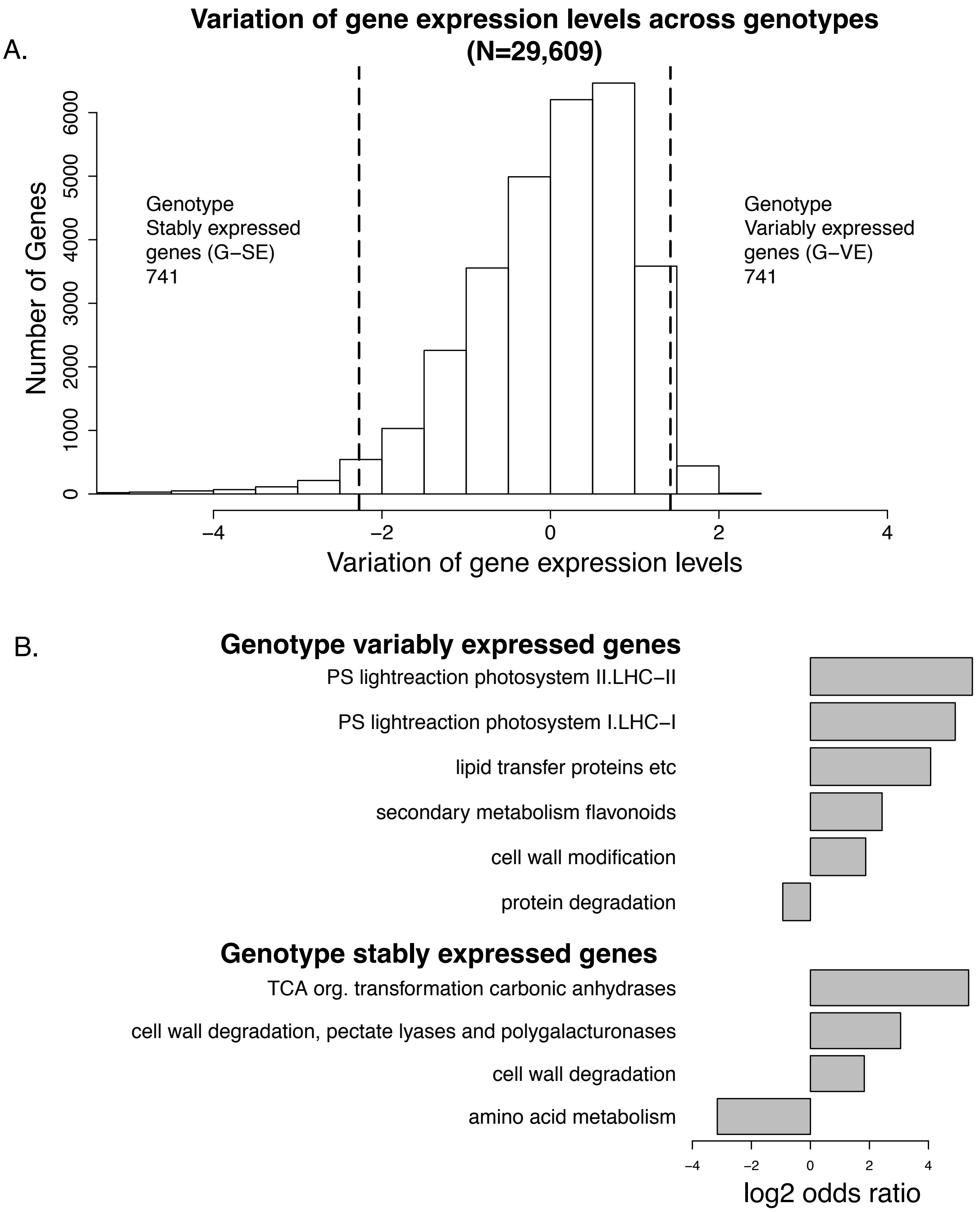

Figure S2. Identification and functional enrichment of G-VE and G-SE genes. a) Histogram of expression variation across tissues; dashed line corresponds to upper and lower 2.5% percentiles of the distribution. b) Functional enrichment of significant categories within G-VE and G-SE genes.

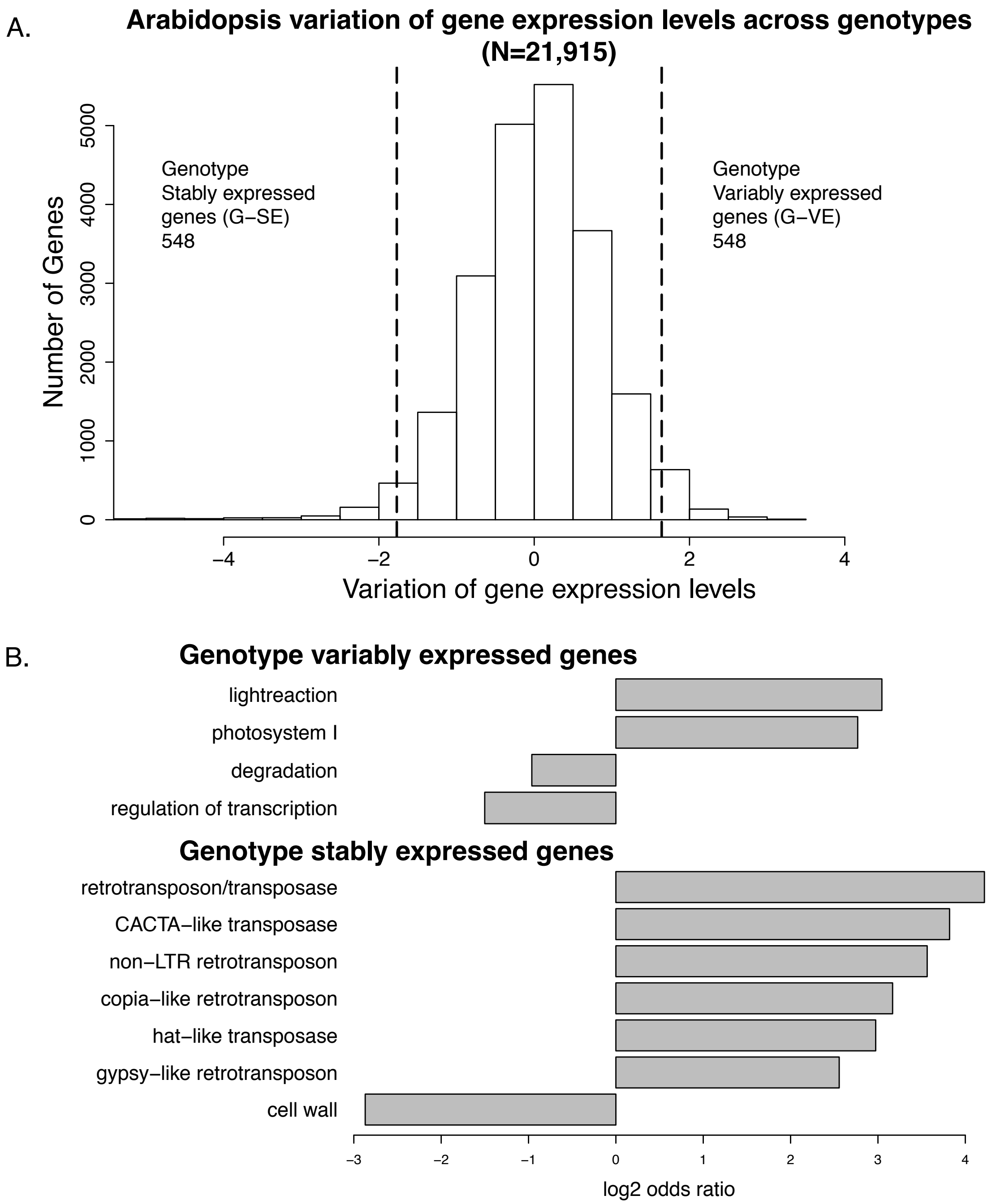

Figure S3. Identification and functional enrichment of G-VE and G-SE genes in Arabidopsis. a) Histogram of expression variation across tissues; dashed line corresponds to upper and lower 2.5% percentiles of the distribution. b) Functional enrichment of significant categories within G-VE and G-SE genes.

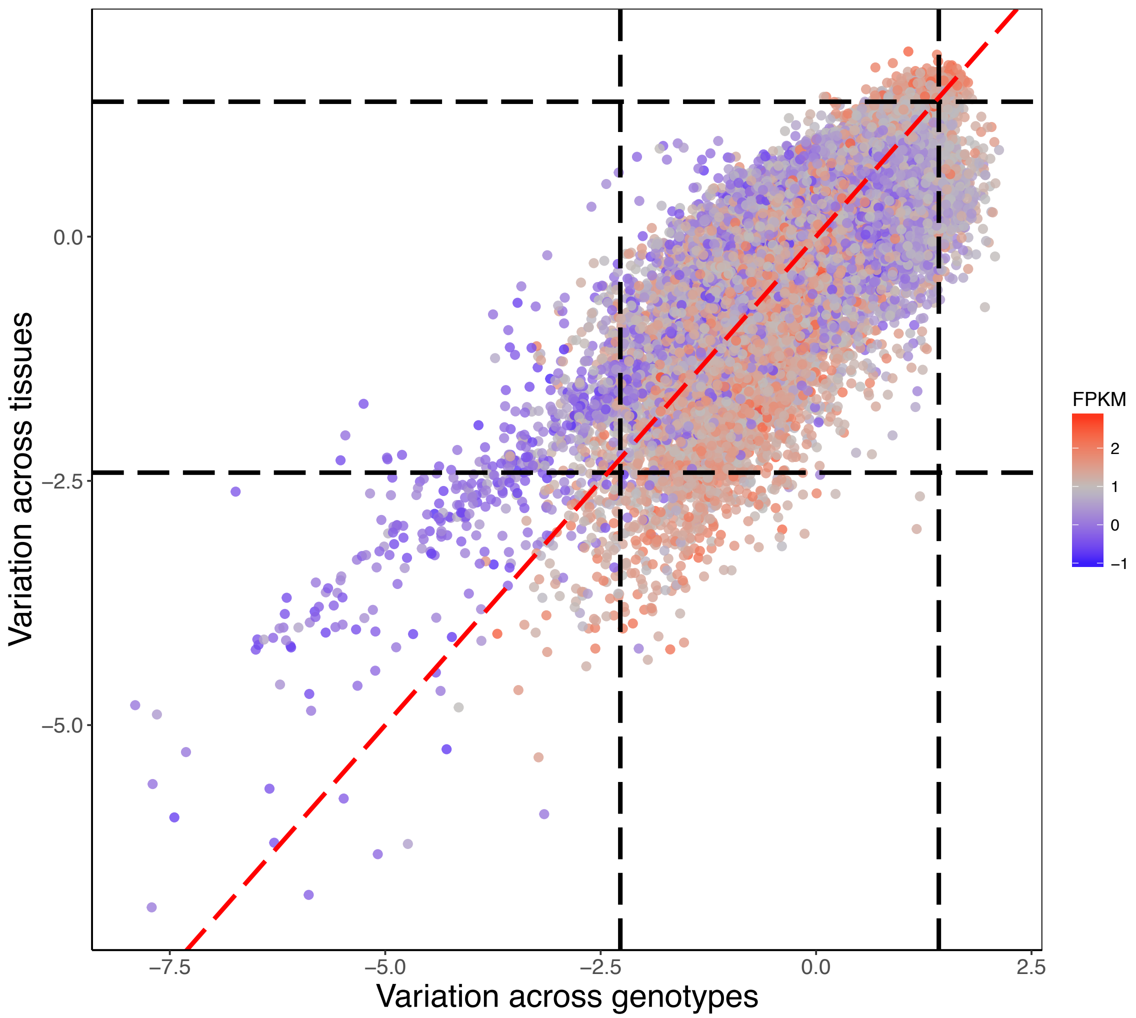

Figure S4. Scatterplot of variation in expression across genotypes and tissues. Colors designate expression levels, with blue the lowest and red the highest; the red dashed line is the diagonal, black dashed lines corresponded to upper and lower 2.5% percentiles for variation in expression.

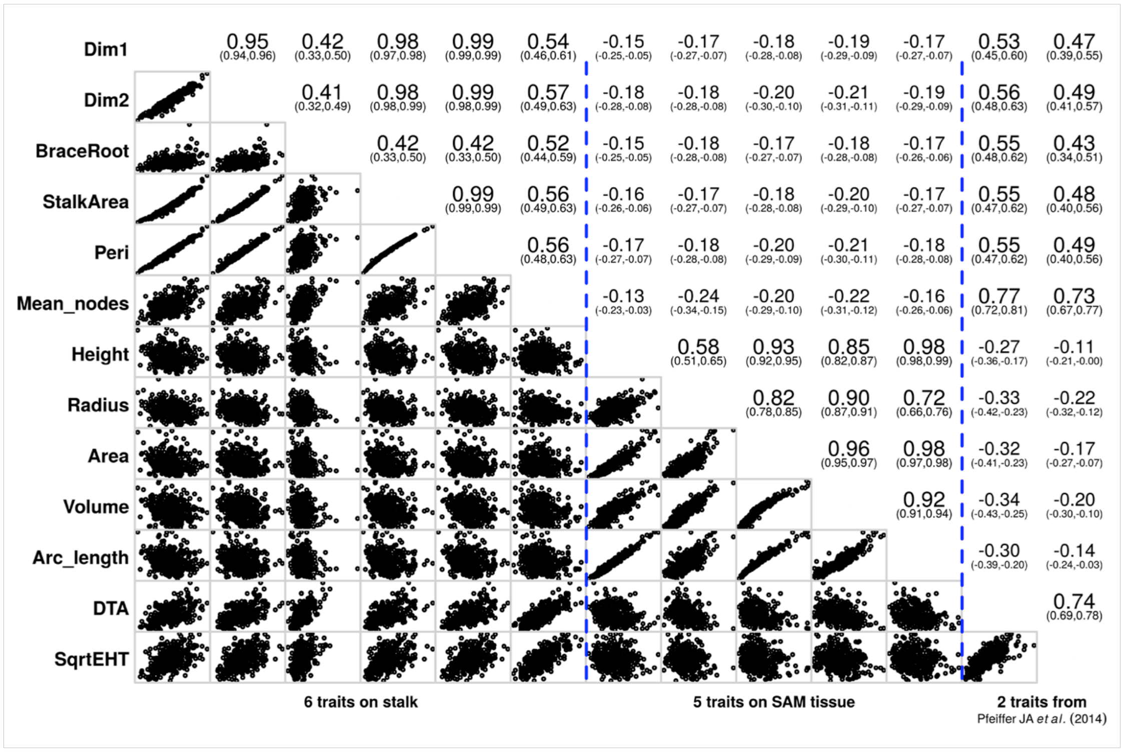

Figure S5. Correlations among 13 traits.

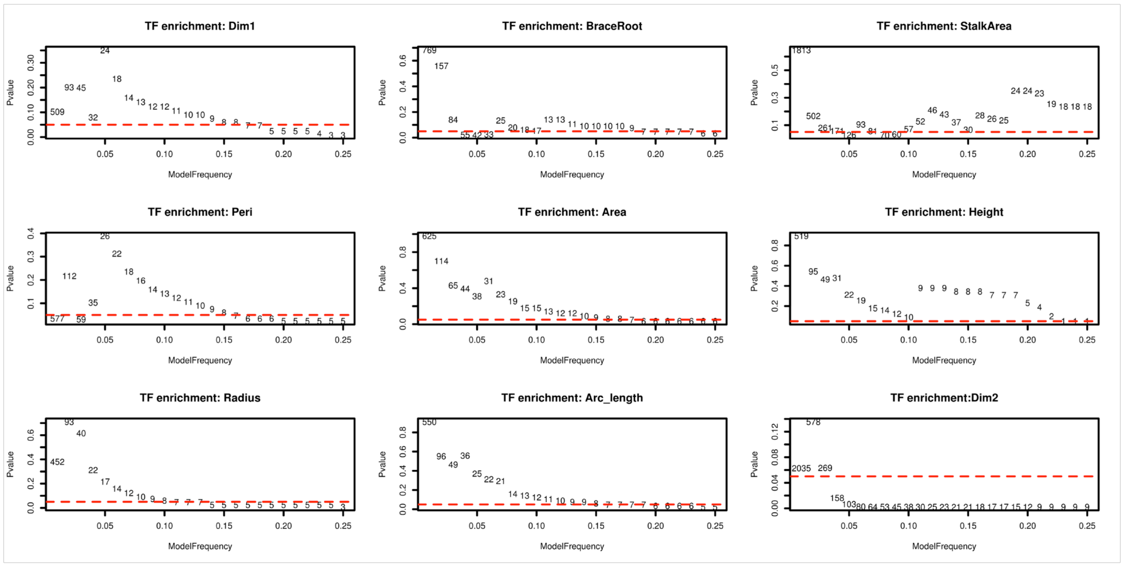

Figure S6. Enrichment testing for eRD genes among 9 traits. This figure shows the enrichments of TFs among eRD genes for various traits at various model frequencies. The number of eRD genes above indicated model frequencies cutoffs are indicated within each plot. The red dashed lines indicate p-values of 0.05.

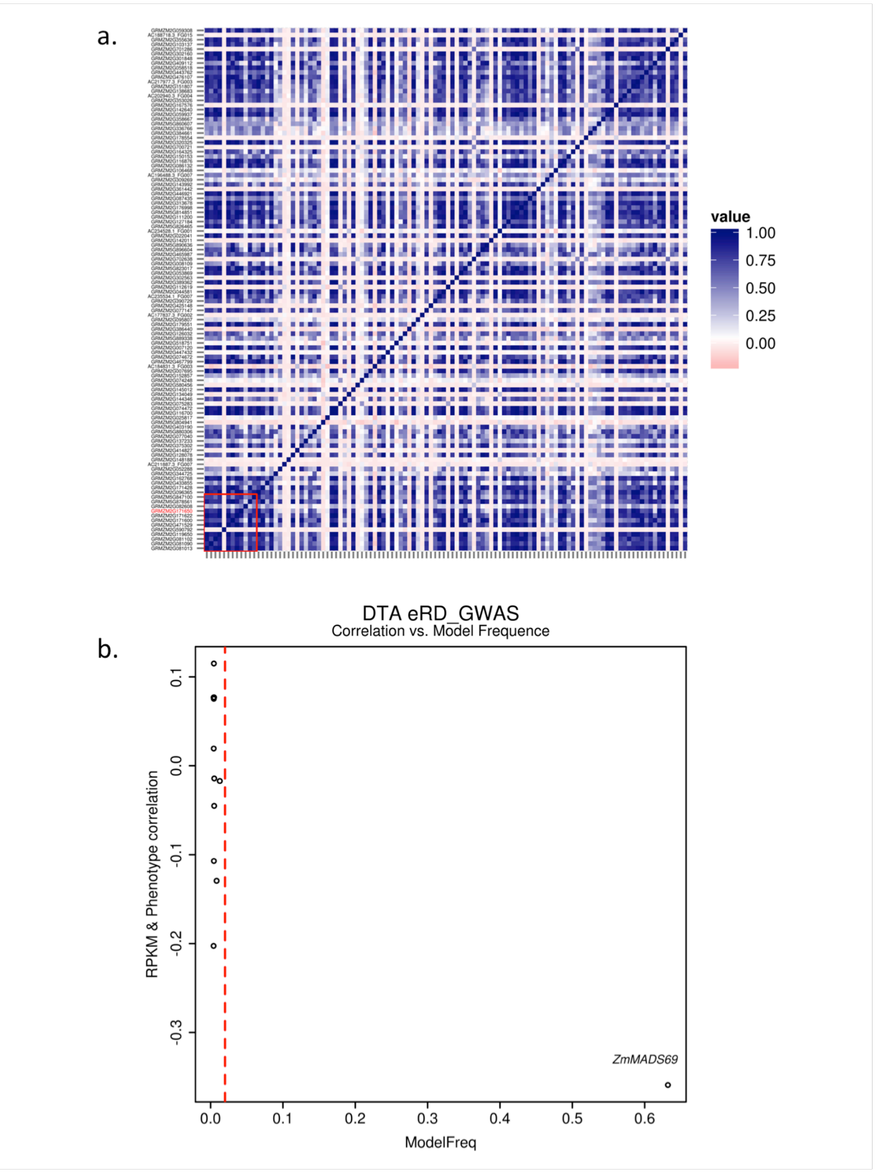

Figure S7. The effect of expression LD on eRD-GWAS. a) A heatmap showing correlation values among RPKM values for 112 genes. The red rectangle is a 1 Mb window centered on the physical position of the ZmMADS69 gene. The remainder of the figure contains 100 randomly chosen genes. b) Relationship between model frequencies from eRD-GWAS for the DTA trait and the correlation between RPKM values of genes and the DTA trait within the 1 Mb window.

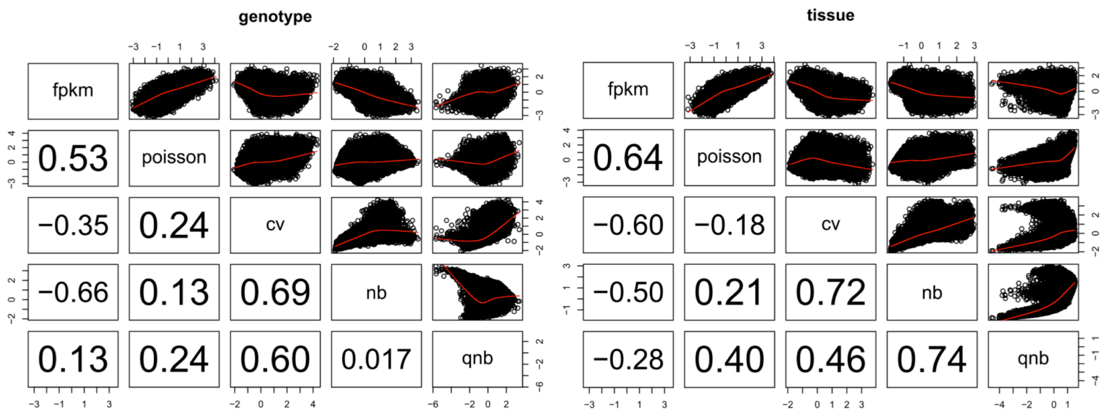

Figure S8. Selection of parameters for measuring expression variation. Pairwise correlations between expression levels (FPKM), the over-dispersion parameter of Poisson (poisson), Coefficient of Variation (cv), dispersion of Negative Binomial models (nb) and over-dispersion parameters of Negative Binomial models (qnb) across genotypes (left panel) and tissues (right panel).
